# Crystal structure of bis­(*N*,*N*,*N*′,*N*′-tetra­methyl­guanidinium) tetra­chlorido­cuprate(II)

**DOI:** 10.1107/S2056989016010161

**Published:** 2016-06-24

**Authors:** Mamadou Ndiaye, Abdoulaye Samb, Libasse Diop, Thierry Maris

**Affiliations:** a Laboratoire des Produits Naturels, Département de Chimie, Faculté des Sciences et Techniques, Université Cheikh Anta Diop, Dakar, Senegal; b Laboratoire de Chimie Minérale et Analytique, Département de Chimie, Faculté des Sciences et Techniques, Université Cheikh Anta Diop, Dakar, Senegal; c Département de Chimie, Université de Montréal, 2900 Boulevard Édouard-Montpetit, Montréal, Québec, H3C 3J7, Canada

**Keywords:** crystal structure, organic–inorganic hybrid salt, tetra­methyl­guanidinium, tetra­chlorido­cuprate(II), τ_4_ index

## Abstract

In the structure of the title salt, (C_5_H_14_N_3_)_2_[CuCl_4_], the Cu^II^ atom in the anion lies on a twofold rotation axis. The tetra­chlorido­cuprate(II) anion adopts a flattened tetra­hedral coordination environment and inter­acts electrostatically with the tetra­methyl­guanidinium cation. The crystal packing is additionally consolidated through N—H⋯Cl and C—H⋯Cl hydrogen bonds, resulting in a three-dimensional network structure.

## Abstract 2

The crystal structure of bis­(tetra­methyl­guanidinium) tetra­chlorido­cuprate(II) contains distorted tetra­hedral [CuCl_4_]^2−^ anions and tetra­methyl­guanidinium cations held together through N—H⋯Cl and C—H⋯Cl hydrogen bonds.

## Chemical context   

The title compound belongs to the series of hybrid organic–inorganic materials of general formula *A*_2_[*MX*_4_] where *A* is an organic cation, *M* a divalent transition metal and *X* a halide. The copper representatives of these families have been extensively studied for their magnetic, dielectric and fluorescent properties in relation to their solid-state structures (Halvorson *et al.*, 1990 ▸). Recent studies include examination of polymorphism in relation to electrostatic properties (Awwadi & Haddad, 2012 ▸) or thermochroism (Aldrich *et al.*, 2016 ▸), sometimes in relation to phase transitions (Kelley *et al.*, 2015 ▸).
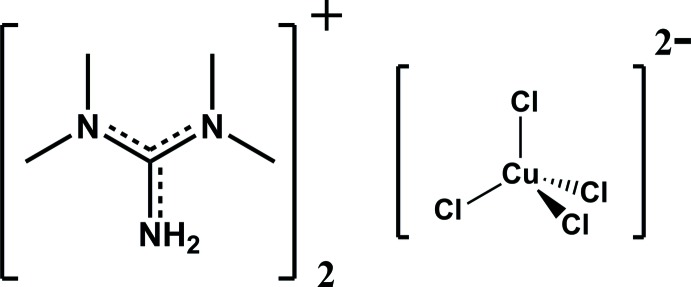


Following our report on the crystal structure of bis-tetra­methyl­guanidinium tri­chlorido­cadmate (Ndiaye *et al.*, 2016 ▸), we have investigated the inter­actions between tetra­methyl­guanidine and CuCl_2_·2H_2_O which has yielded the title salt, (C_5_H_14_N_3_)_2_[CuCl_4_], (I).

## Structural commentary   

**Figure 1:**
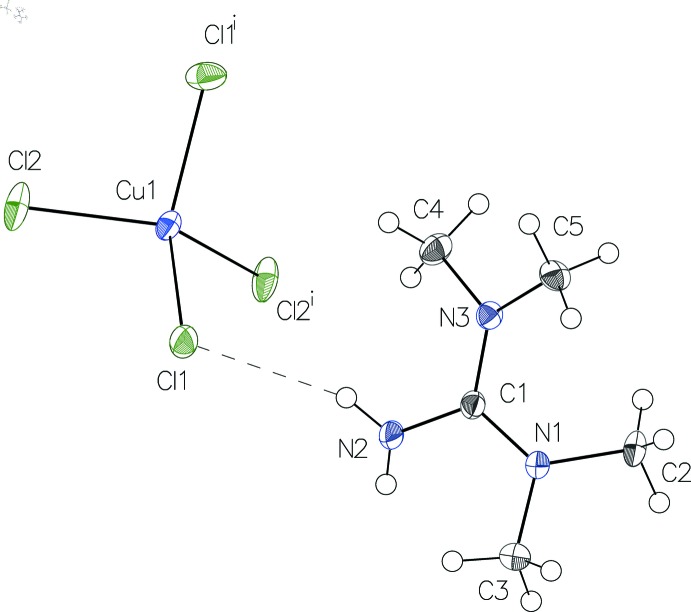
The structures of the mol­ecular entities in (I), drawn with displacement parameters at the 50% probability level. The N—H⋯Cl hydrogen bond is indicated by a dashed line. [Symmetry code: (i) −*x* + 1, *y*, −*z* + 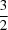.]

The asymmetric unit of (I) contains a complete *N*,*N*,*N*′,*N*′-tetra­methyl­guanidinium cation and half of a [CuCl_4_]^2−^ anion held together by an N—H⋯Cl hydrogen bond (Fig. 1 ▸). In the anion, the Cu—Cl distances range from 2.2396 (4) Å to 2.2557 (4) Å. They are shorter than those usually found in tetra­chlorido­cuprate(II) anions with a square-planar configuration (Guo *et al.*, 2015 ▸). The distortion of the flattened tetra­chlorido­cuprate(II) anion in (I) from the ideal tetra­hedral configuration can be asserted by the values of the two *trans* Cl—Cu—Cl angles, 135.62 (3)° and 133.31 (3)°. These two angles can also be used to calculate the *τ*_4_ geometry index developed by Yang *et al.* (2007 ▸) for complexes with coordination number four to qu­antify such a distortion. The *τ*_4_ parameter is defined as [360 - (*α*+*β*)] / 141 where *α* and *β* are the two largest Cl—Cu—Cl angles. A *τ*_4_ index value of 1 corresponds to an ideal tetra­hedral configuration while a value of 0 is for a perfect square-planar configuration. Here the value obtained (0.65) indicates a ‘see-saw’ (bis­phenoidal) configuration with point group symmetry 2.

In the organic cation, the C—N distances in the central CN_3_ unit [1.332 (2), 1.335 (2) and 1.342 (2) Å] are consistent with a partial double-bond character and a positive charge delocalization, as usually found in structures involving tetra­methyl­guanidinium cations. The central core of the cation has an almost planar–trigonal geometry, as reflected by the values for the three N—C—N angles close to 120° and the r.m.s deviation from the least-squares plane calculated for atoms C1, N1, N2 and N3 that is only 0.0006 Å. The di­methyl­ammonium groups are twisted by 29.38 (16)° (C2, C3) and 25.08 (16)° (C4, C5) with respect to this plane.

## Supra­molecular features   

**Figure 2:**
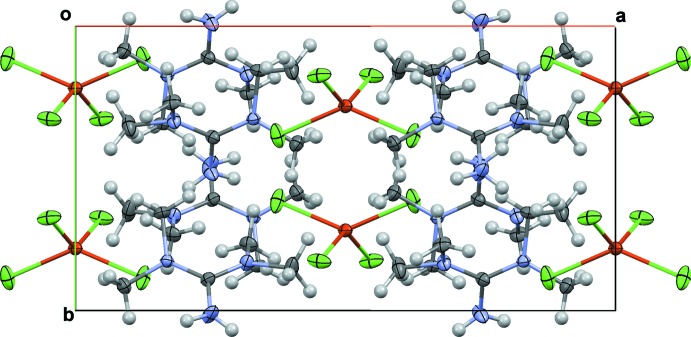
Packing diagram of (I) viewed along [001].

Anions and cations are connected through electrostatic inter­actions and *via* classical N—H⋯Cl hydrogen bonds involving atom Cl1 whereby only one of the H atoms of the amine group is involved; the remaining H atom has no acceptor atom (Fig. 1 ▸, Table 1 ▸). In addition, each Cl atom of the anion is engaged in three C—H⋯Cl hydrogen bonds, leading to the formation of a three-dimensional network structure (Fig. 2 ▸, Table 1 ▸).

## Database survey   

A search in the Cambridge Structural Database (Version 5.37 with two updates; Groom *et al.*, 2016 ▸) for isolated tetra­chlorido­cuprate(II) anions without disorder returned 342 hits for a total of 389 fragments. The configurations of these fragments were analysed using the *τ*_4_ index as described above. Around 60 of these (15%) have a *τ*_4_ index value less than 0.1, including 29 that have a *τ*_4_ index of 0 (ideal square-planar configuration). Only four were found to have a configuration close to the ideal tetra­hedral one with a *τ*_4_ index value larger than 0.9. A large number of fragments (72%) has a geometry index *τ*_4_ value in the 0.6–0.8 range and feature a bis­phenoidal configuration as found for (I). An analysis with the modified version of the *τ*_4_ index [*τ*_4_′, as defined by Okuniewski *et al.* (2015 ▸)] gives a similar distribution with only minor variation.

The title compound is isostructural with bis­(*N*,*N*,*N*′,*N*′-tetra­methyl­guanidinium) tetra­bromido­nickelate(II) (Jones & Thonnessen, 2006 ▸) and shows similarities in terms of the space-group and cell parameters with tetra­methyl­guanidinium bis­ulfite (Heldebrant *et al.*, 2009 ▸).

## Synthesis and crystallization   

Yellowish-green crystals were obtained by mixing in stoichiometric amounts tetra­methyl­guanidine with CuCl_2_·2H_2_O in ethanol.

## Refinement   

Crystal data, data collection and structure refinement details are summarized in Table 2 ▸. All hydrogen atoms were located from a Fourier difference map and were refined freely.

## Supplementary Material

Crystal structure: contains datablock(s) I. DOI: 10.1107/S2056989016010161/wm5303sup1.cif

Structure factors: contains datablock(s) I. DOI: 10.1107/S2056989016010161/wm5303Isup2.hkl

CCDC reference: 1486986

Additional supporting information: crystallographic information; 3D view; checkCIF report

## Figures and Tables

**Table 1:** Hydrogen-bond geometry (Å, °)

*D*—H⋯*A*	*D*—H	H⋯*A*	*D*⋯*A*	*D*—H⋯*A*
C4—H4*B*⋯Cl2^i^	0.95 (2)	2.77 (2)	3.5902 (19)	145.3 (18)
C2—H2*C*⋯Cl1^ii^	0.98 (3)	2.90 (3)	3.745 (2)	144.5 (18)
C2—H2*D*⋯Cl1^iii^	0.91 (2)	2.91 (2)	3.818 (2)	173.3 (19)
C3—H3*B*⋯Cl2^iv^	0.99 (3)	2.82 (3)	3.793 (2)	168 (2)
C5—H5*C*⋯Cl2^v^	0.93 (3)	2.80 (3)	3.5992 (18)	144.9 (19)
C2—H2*E*⋯Cl2^vi^	0.96 (3)	2.85 (3)	3.6491 (18)	140.5 (19)
N2—H2*B*⋯Cl1	0.86 (3)	2.53 (3)	3.3417 (16)	157 (2)

Symmetry codes: (i) 
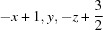
; (ii) 
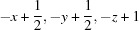
; (iii) 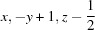; (iv) 
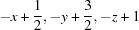
; (v) 
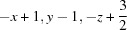
; (vi) 
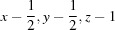
.

**Table 2:** Experimental details

Crystal data	
Chemical formula	(C_5_H_14_N_3_)_2_[CuCl_4_]
*M*_r_	437.72
Crystal system, space group	Monoclinic, *C*2/*c*
Temperature (K)	100
*a*, *b*, *c* (Å)	18.9274 (5), 8.2441 (2), 14.8654 (4)
β (°)	124.165 (1)
*V* (Å^3^)	1919.28 (9)
*Z*	4
Radiation type	Ga *K*α, λ = 1.34139 Å
μ (mm^−1^)	9.51
Crystal size (mm)	0.16 × 0.10 × 0.06

Data collection	
Diffractometer	Bruker Venture Metaljet
Absorption correction	Multi-scan (*SADABS*; Krause *et al.*, 2015 ▸)
*T*_min_, *T*_max_	0.449, 0.752
No. of measured, independent and observed [*I* > 2σ(*I*)] reflections	14222, 2208, 2178
*R*_int_	0.036
(sin θ/λ)_max_ (Å^−1^)	0.650

Refinement	
*R*[*F* ^2^ > 2σ(*F* ^2^)], *wR*(*F* ^2^), *S*	0.028, 0.071, 1.11
No. of reflections	2208
No. of parameters	152
H-atom treatment	All H-atom parameters refined
Δρ_max_, Δρ_min_ (e Å^−3^)	0.84, −0.35

Computer programs: *APEX2* and *SAINT* (Bruker, 2014 ▸), *SHELXT* (Sheldrick, 2015*a* ▸), *SHELXL2014* (Sheldrick, 2015*b* ▸), *OLEX2* (Dolomanov *et al.*, 2009 ▸), *Mercury* (Macrae *et al.*, 2008 ▸) and *publCIF* (Westrip, 2010 ▸).

## supplementary crystallographic information

### Crystal data

**Table:**

(C_5_H_14_N_3_)_2_[CuCl_4_]	*F*(000) = 908
*M**_r_* = 437.72	*D*_x_ = 1.515 Mg m^−^^3^
Monoclinic, *C*2/*c*	Ga *K*α radiation, λ = 1.34139 Å
*a* = 18.9274 (5) Å	Cell parameters from 9968 reflections
*b* = 8.2441 (2) Å	θ = 4.9–60.7°
*c* = 14.8654 (4) Å	µ = 9.51 mm^−^^1^
β = 124.165 (1)°	*T* = 100 K
*V* = 1919.28 (9) Å^3^	Block, clear yellowish green
*Z* = 4	0.16 × 0.10 × 0.06 mm

### Data collection

**Table:**

Bruker Venture Metaljet diffractometer	2208 independent reflections
Radiation source: Metal Jet, Gallium Liquid Metal Jet Source	2178 reflections with *I* > 2σ(*I*)
Helios MX Mirror Optics monochromator	*R*_int_ = 0.036
Detector resolution: 10.24 pixels mm^-1^	θ_max_ = 60.7°, θ_min_ = 4.9°
ω and φ scans	*h* = −24→24
Absorption correction: multi-scan (*SADABS*; Krause *et al.*, 2015)	*k* = −10→10
*T*_min_ = 0.449, *T*_max_ = 0.752	*l* = −16→19
14222 measured reflections	

### Refinement

**Table:**

Refinement on *F*^2^	Primary atom site location: structure-invariant direct methods
Least-squares matrix: full	Hydrogen site location: difference Fourier map
*R*[*F*^2^ > 2σ(*F*^2^)] = 0.028	All H-atom parameters refined
*wR*(*F*^2^) = 0.071	*w* = 1/[σ^2^(*F*_o_^2^) + (0.0315*P*)^2^ + 2.7253*P*] where *P* = (*F*_o_^2^ + 2*F*_c_^2^)/3
*S* = 1.11	(Δ/σ)_max_ = 0.001
2208 reflections	Δρ_max_ = 0.84 e Å^−^^3^
152 parameters	Δρ_min_ = −0.35 e Å^−^^3^
0 restraints	

### Special details

**Table:**

Experimental. X-ray crystallographic data for I were collected from a single crystal sample, which was mounted on a loop fiber. Data were collected using a Bruker Venture diffractometer equipped with a Photon 100 CMOS Detector, a Helios MX optics and a Kappa goniometer. The crystal-to-detector distance was 4.0 cm, and the data collection was carried out in 1024 x 1024 pixel mode.
Geometry. All esds (except the esd in the dihedral angle between two l.s. planes) are estimated using the full covariance matrix. The cell esds are taken into account individually in the estimation of esds in distances, angles and torsion angles; correlations between esds in cell parameters are only used when they are defined by crystal symmetry. An approximate (isotropic) treatment of cell esds is used for estimating esds involving l.s. planes.

### Fractional atomic coordinates and isotropic or equivalent isotropic displacement parameters (Å^2^)

**Table:**

	*x*	*y*	*z*	*U*_iso_*/*U*_eq_	
N1	0.18244 (9)	0.33219 (19)	0.30111 (11)	0.0202 (3)	
N2	0.24981 (11)	0.5129 (2)	0.44495 (13)	0.0277 (4)	
N3	0.32977 (9)	0.32615 (17)	0.42260 (11)	0.0164 (3)	
C1	0.25428 (10)	0.3903 (2)	0.38962 (13)	0.0173 (3)	
C2	0.17917 (12)	0.2676 (2)	0.20725 (14)	0.0215 (3)	
C3	0.09950 (12)	0.3500 (3)	0.28512 (17)	0.0314 (4)	
C4	0.40978 (11)	0.4132 (2)	0.49381 (15)	0.0235 (4)	
C5	0.33922 (12)	0.1564 (2)	0.40153 (15)	0.0208 (3)	
Cu1	0.5000	0.71829 (4)	0.7500	0.01698 (11)	
Cl1	0.37443 (3)	0.61496 (7)	0.70754 (4)	0.03106 (13)	
Cl2	0.54835 (3)	0.82595 (5)	0.91317 (3)	0.02989 (13)	
H4A	0.4363 (15)	0.380 (3)	0.569 (2)	0.030 (6)*	
H5A	0.2894 (16)	0.099 (3)	0.3780 (19)	0.029 (6)*	
H4B	0.4000 (14)	0.526 (3)	0.4851 (18)	0.024 (5)*	
H2C	0.1702 (16)	0.150 (3)	0.2047 (19)	0.030 (6)*	
H2D	0.2285 (15)	0.291 (3)	0.2132 (18)	0.022 (5)*	
H3A	0.0693 (18)	0.248 (4)	0.260 (2)	0.040 (7)*	
H2A	0.2102 (18)	0.568 (4)	0.418 (2)	0.037 (7)*	
H3B	0.0655 (19)	0.432 (4)	0.228 (2)	0.050 (8)*	
H5B	0.3539 (15)	0.147 (3)	0.349 (2)	0.028 (6)*	
H5C	0.3857 (16)	0.111 (3)	0.465 (2)	0.028 (6)*	
H2E	0.1316 (16)	0.319 (3)	0.143 (2)	0.028 (6)*	
H4C	0.4454 (16)	0.389 (3)	0.468 (2)	0.033 (6)*	
H2B	0.2934 (17)	0.536 (3)	0.509 (2)	0.035 (6)*	
H3C	0.1050 (17)	0.373 (3)	0.352 (2)	0.040 (7)*	

### Atomic displacement parameters (Å^2^)

**Table:**

	*U*^11^	*U*^22^	*U*^33^	*U*^12^	*U*^13^	*U*^23^
N1	0.0176 (6)	0.0242 (7)	0.0155 (6)	0.0023 (6)	0.0072 (5)	−0.0054 (6)
N2	0.0225 (7)	0.0289 (8)	0.0186 (7)	0.0092 (7)	0.0036 (6)	−0.0097 (6)
N3	0.0174 (6)	0.0135 (6)	0.0163 (6)	−0.0003 (5)	0.0083 (5)	−0.0008 (5)
C1	0.0196 (7)	0.0167 (7)	0.0130 (7)	0.0028 (6)	0.0075 (6)	−0.0001 (6)
C2	0.0273 (9)	0.0215 (9)	0.0134 (8)	−0.0026 (7)	0.0102 (7)	−0.0044 (6)
C3	0.0182 (8)	0.0432 (12)	0.0264 (9)	0.0061 (8)	0.0086 (7)	−0.0100 (9)
C4	0.0194 (8)	0.0228 (9)	0.0223 (8)	−0.0040 (7)	0.0079 (7)	−0.0029 (7)
C5	0.0234 (8)	0.0132 (7)	0.0253 (9)	0.0028 (7)	0.0135 (7)	0.0000 (7)
Cu1	0.01872 (18)	0.01483 (18)	0.01209 (17)	0.000	0.00542 (14)	0.000
Cl1	0.0201 (2)	0.0510 (3)	0.0243 (2)	−0.00970 (18)	0.01383 (17)	−0.01532 (19)
Cl2	0.0501 (3)	0.0180 (2)	0.01273 (19)	−0.00519 (18)	0.01222 (18)	−0.00224 (14)

### Geometric parameters (Å, º)

**Table:**

N1—C1	1.342 (2)	C3—H3B	0.99 (3)
N1—C2	1.462 (2)	C3—H3C	0.96 (3)
N1—C3	1.457 (2)	C4—H4A	0.97 (3)
N2—C1	1.335 (2)	C4—H4B	0.95 (2)
N2—H2A	0.77 (3)	C4—H4C	0.97 (3)
N2—H2B	0.86 (3)	C5—H5A	0.93 (3)
N3—C1	1.332 (2)	C5—H5B	0.96 (2)
N3—C4	1.459 (2)	C5—H5C	0.93 (3)
N3—C5	1.467 (2)	Cu1—Cl1^i^	2.2557 (4)
C2—H2C	0.98 (3)	Cu1—Cl1	2.2557 (4)
C2—H2D	0.91 (2)	Cu1—Cl2^i^	2.2396 (4)
C2—H2E	0.96 (3)	Cu1—Cl2	2.2396 (4)
C3—H3A	0.96 (3)		
			
C1—N1—C2	122.85 (14)	H3A—C3—H3B	108 (2)
C1—N1—C3	121.97 (14)	H3A—C3—H3C	105 (2)
C3—N1—C2	114.63 (14)	H3B—C3—H3C	113 (2)
C1—N2—H2A	120 (2)	N3—C4—H4A	110.4 (14)
C1—N2—H2B	119.9 (17)	N3—C4—H4B	110.0 (14)
H2A—N2—H2B	120 (3)	N3—C4—H4C	106.3 (15)
C1—N3—C4	122.24 (14)	H4A—C4—H4B	112 (2)
C1—N3—C5	122.31 (14)	H4A—C4—H4C	112 (2)
C4—N3—C5	115.01 (14)	H4B—C4—H4C	106 (2)
N2—C1—N1	119.64 (15)	N3—C5—H5A	110.2 (15)
N3—C1—N1	120.38 (15)	N3—C5—H5B	111.8 (15)
N3—C1—N2	119.98 (15)	N3—C5—H5C	109.2 (15)
N1—C2—H2C	108.1 (14)	H5A—C5—H5B	110 (2)
N1—C2—H2D	109.8 (14)	H5A—C5—H5C	111 (2)
N1—C2—H2E	107.2 (15)	H5B—C5—H5C	104 (2)
H2C—C2—H2D	111 (2)	Cl1—Cu1—Cl1^i^	135.62 (3)
H2C—C2—H2E	110 (2)	Cl2—Cu1—Cl1	100.265 (17)
H2D—C2—H2E	110 (2)	Cl2^i^—Cu1—Cl1	96.958 (19)
N1—C3—H3A	109.1 (16)	Cl2—Cu1—Cl1^i^	96.959 (19)
N1—C3—H3B	109.1 (17)	Cl2^i^—Cu1—Cl1^i^	100.264 (17)
N1—C3—H3C	111.8 (16)	Cl2—Cu1—Cl2^i^	133.31 (3)
			
C2—N1—C1—N2	146.94 (18)	C4—N3—C1—N1	159.80 (16)
C2—N1—C1—N3	−33.3 (3)	C4—N3—C1—N2	−20.4 (2)
C3—N1—C1—N2	−24.1 (3)	C5—N3—C1—N1	−28.2 (2)
C3—N1—C1—N3	155.67 (18)	C5—N3—C1—N2	151.61 (17)

Symmetry code: (i) −*x*+1, *y*, −*z*+3/2.

### Hydrogen-bond geometry (Å, º)

**Table:**

*D*—H···*A*	*D*—H	H···*A*	*D*···*A*	*D*—H···*A*
C4—H4*B*···Cl2^i^	0.95 (2)	2.77 (2)	3.5902 (19)	145.3 (18)
C2—H2*C*···Cl1^ii^	0.98 (3)	2.90 (3)	3.745 (2)	144.5 (18)
C2—H2*D*···Cl1^iii^	0.91 (2)	2.91 (2)	3.818 (2)	173.3 (19)
C3—H3*B*···Cl2^iv^	0.99 (3)	2.82 (3)	3.793 (2)	168 (2)
C5—H5*C*···Cl2^v^	0.93 (3)	2.80 (3)	3.5992 (18)	144.9 (19)
C2—H2*E*···Cl2^vi^	0.96 (3)	2.85 (3)	3.6491 (18)	140.5 (19)
N2—H2*B*···Cl1	0.86 (3)	2.53 (3)	3.3417 (16)	157 (2)

Symmetry codes: (i) −*x*+1, *y*, −*z*+3/2; (ii) −*x*+1/2, −*y*+1/2, −*z*+1; (iii) *x*, −*y*+1, *z*−1/2; (iv) −*x*+1/2, −*y*+3/2, −*z*+1; (v) −*x*+1, *y*−1, −*z*+3/2; (vi) *x*−1/2, *y*−1/2, *z*−1.
